# Does online food shopping boost dietary diversity? Application of an endogenous switching model with a count outcome variable

**DOI:** 10.1186/s40100-022-00239-2

**Published:** 2022-12-05

**Authors:** Wanglin Ma, Puneet Vatsa, Hongyun Zheng, Yanzhi Guo

**Affiliations:** 1 Department of Global Value Chains and Trade, Faculty of Agribusiness and Commerce, Lincoln University, Christchurch, New Zealand; 2 College of Economics & Management, Huazhong Agricultural University, Wuhan, China; 3 Institute of Food and Nutrition Development, Ministry of Agriculture and Rural Affairs, Beijing, China

**Keywords:** Online food shopping, Dietary diversity, Endogenous switching, Rural China, D12, E21, M14, C24

## Abstract

Increasingly, rural households in developing countries are shopping for food online, and the COVID-19 pandemic has accelerated this trend. In parallel, dietary guidelines worldwide recommend eating a balanced and healthy diet. With this in mind, this study explores whether online food shopping boosts dietary diversity—defined as the number of distinct food groups consumed—among rural households in China. Because people choose to shop for food online, it is important to account for the self-selection bias inherent in online food shopping. Accordingly, we estimate the treatment effects of online food shopping on dietary diversity using the endogenous switching model with a count outcome variable. The results indicate that online food shopping increases dietary diversity by 7.34%. We also find that education, asset ownership, and knowing the government’s dietary guidelines are the main factors driving rural households’ decisions to shop for food online.

## Introduction

Dietary guidelines worldwide encourage people to eat a healthy and balanced diet comprising various food groups. Dietary diversity promotes health, well-being, and food security.^1^ Numerous studies have confirmed the salubrious effects of consuming a diverse diet. It is associated with longer life spans and healthspans (Miyamoto et al. 2019; Lagström et al. 2020; Otsuka et al. 2020), lower incidence of ischemic heart disease, type 2 diabetes and metabolic syndrome (Mozaffari et al. 2021; Sezaki et al. 2021; Tosi and Rettaroli 2022), and reduced depression, stress and anxiety (Poorrezaeian et al. 2017; Jiang et al. 2018; Freire and Rudkin 2019). These findings underscore why promoting dietary diversity should be an integral part of multifaceted public health initiatives. Nevertheless, despite mounting evidence for the benefits of dietary diversity, why it is increasingly slowly and what influences it remains unclear. To promote dietary diversity, one must understand the social and economic context in which people buy and consume food. Accordingly, this paper is devoted to analyzing the role of online shopping, a fast-growing industry changing the buying and consumption patterns globally, on dietary diversity.

A growing number of studies have investigated various factors driving overall household nutrition intake and dietary diversity. These include, for example, women’s employment (Larson et al. 2019; Rao et al. 2019; Kassie et al. 2020; Nikiema and Sakurai 2021), adoption of information and communication technologies (ICTs) (e.g., Internet use, mobile phone use) (Parlasca et al. 2020; Ankrah Twumasi et al. 2021), adoption of climate-smart agricultural practices (Shahzad and Abdulai 2020; Issahaku and Abdulai 2020), non-farm income (Pritchard et al. 2019; Rahman and Mishra 2020), access to credit (Islam et al. 2016; Annim and Frempong 2018), agricultural commercialization (Loos and Zeller 2014), contract farming (Debela et al. 2022), and production diversification (Jones et al. 2014; Dillon et al. 2015; Ecker 2018; Sibhatu and Qaim 2018; Ayenew et al. 2018; Chegere and Stage 2020; Aweke et al. 2020). Rural households usually diversify their diets through diversifying crops and livestock in agricultural production. However, this can be challenging, as each household can only cultivate a limited range of crops due to regional climatic conditions and limited land size.

Some studies have compared the differences between farm production and market access in improving rural households’ dietary diversity (Koppmair et al. 2017; Zanello et al. 2019; Muthini et al. 2020; Madzorera et al. 2021; Olabisi et al. 2021). In general, they have found that market access plays a larger role in improving dietary diversity relative to production diversification. By estimating data from smallholder farm households in Indonesia, Kenya, Ethiopia, and Malawi, Sibhatu et al. (2015) found that production diversity significantly increases dietary diversity in some situations; they argued that improving smallholder farmers’ access to markets is a more effective strategy for improving dietary diversity. The finding was further supported by Olabisi et al. (2021), who reported that Nigerian households buying all their food from markets had higher dietary diversity scores compared with those producing a greater share of their own food.

Although access to traditional local markets helps rural households diversify their food purchases to some extent, the emergence and development of online shopping platforms have made it easier to discover and purchase a wide range of products, including food items, from anywhere in the world. People can purchase food items on sellers’ websites or apps using Internet payment or mobile payments (e.g., WeChat pay, Alipay, or credit cards) and have them delivered to their doorsteps or nearby collection centers. Online food shopping allows consumers to access both national and global markets and helps them save time that otherwise would have been spent commuting to retail stores and lining up at checkout tills in traditional brick and mortar retail stores. Furthermore, online food shopping allows customers to browse innovative online catalogues and discover new products, discounts and deals, and build customized shopping carts comprising their favorite items. The conveniences and advantages of online shopping may encourage rural customers to purchase food online, which may increase their dietary diversity.

Although there is a growing literature on customers' preferences for and attitudes toward online food shopping (Heng et al. 2018; Bryła 2018; Wang et al. 2019; Balachandran et al. 2020; Liu and Lin 2020; Kim et al. 2021), the association between online food shopping and dietary diversity remains unexamined. This paper fills this gap in the literature. Using farm household survey data collected from 947 households in China's Shandong, Guangxi, Henan, and Sichuan provinces, we explore whether online food shopping boosts or inhibits the dietary diversity of rural households in China. Dietary diversity is measured by dietary diversity scores, referring to the number of food groups consumed by rural households in the last 72 h—as such, it is a count variable. Furthermore, it bears emphasizing that rural households decide whether to shop online. This decision is neither imposed on them nor are some households instructed not to shop online (i.e., self-selection). Thus, shopping online is potentially endogenous. With this in mind, we estimate a newly developed endogenous switching model with a count outcome variable. This model simultaneously addresses the potential selection bias and estimates the treatment effects of online food shopping on dietary diversity (Hasebe 2020). It can also help us understand the determinants of online food shopping and factors affecting dietary diversity, respectively, for online food shoppers and non-shoppers. Because rural households tend to lag their urban counterparts in accessing nutritious and diversified foods (Nii et al. 2016; Krishna Bahadur et al. 2018; Rupa et al. 2019; Raj et al. 2020), improving food and nutrition security and dietary diversity of rural households can contribute to overall social welfare.

The rest of this study is structured as follows: Sect. 2 details the econometric model. Section 3 describes the data and definitions of key variables. The empirical results are presented and discussed in Sect. 4. The final section concludes the paper and lays out policy implications.

## Econometric model

Before describing the model, we want to emphasize two points that we have alluded to in Sect. 1. First, people choose to shop or not to shop online (Gao et al. 2020; Zheng and Ma 2021a; Chang and Meyerhoefer 2021). This choice is not imposed on or randomly assigned to them and may be influenced by both observed factors (e.g., age, gender, and education) and unobserved factors (e.g., innate abilities, competence in using technology, and motivations). Thus, self-selection bias should be duly addressed to glean meaningful insights into the links between online food shopping and selection bias. Second, the dependent variable, i.e., dietary diversity, is measured as a count variable. Therefore, it is vitally important to use a modeling approach designed to explain variations in count-dependent variables.

When estimating the impact of a binary treatment variable on a count variable, researchers have used propensity score matching (PSM) (Caliendo and Kopeinig 2008; Abadie and Imbens 2016; Khachatryan et al. 2019), the inverse-probability-weighted regression adjustment (IPWRA) estimator (Manda et al. 2018; Liu et al. 2019; Zheng and Ma 2021b), Poisson regression with endogenous treatment effects (PRETE) (Miranda and Rabe-Hesketh 2006; Bratti and Miranda 2011), and endogenous switching regression for count-dependent variables (henceforth, ESC model) (Hasebe 2020). Among them, PSM and IPWRA are nonparametric approaches that address only the selection bias from observed factors. In contrast, the PRETE model can mitigate selection bias arising from both observed and unobserved factors; however, it only estimates one selection equation and one outcome equation. In this regard, the ESC model is distinctly advantageous: it can mitigate observed and unobserved selection biases, and it simultaneously estimates one selection equation and two outcome equations (Regime 1 and Regime 2) using the maximum likelihood estimator (MLE) as follows (Hasebe 2020):

1 $${\text{Selection}}\;{\text{equation: OFS}}_{i}^{*} = Z_{i}^{{\prime }} \alpha _{i} + \varepsilon _{i} ,\quad {\text{where}}\quad T_{i} = \left\{ {\begin{array}{*{20}c} 1 & {f\;{\text{OFS}}_{i}^{*} > 0} \\ 0 & {{\text{otherwise}}} \\ \end{array} } \right.$$

2a $${\text{Regime 1}}(T_{i} = 1):\quad Y_{1i} = X_{i}^{^{\prime}} \beta_{1i} + \eta_{1i}$$

2b $${\text{Regime 2 }}(T_{i} = 0):\quad Y_{0i} = X_{i}^{^{\prime}} \beta_{0i} + \eta_{0i}$$

where ${\text{OFS}}_{i}^{*}$ represents the probability of household $i$ choosing to shop for food online, and it is observed by $T_{i}$ ($T_{i} = 1$ for online food shoppers and $T_{i} = 0$ for non-shoppers). $Y_{1i}$ and $Y_{0i}$ refer to dietary diversity for online food shoppers and non-shoppers, respectively.$\alpha_{i}$ and $\beta_{ji}$($j = 1,{ }0$) are parameters to be estimated. $\varepsilon_{i}$ and $\eta_{ji}$ ($j = 1,{ }0$) are error terms. $Z_{i}^{^{\prime}}$ is a vector of control variables (e.g., age, gender, asset ownership, and distance to a market) influencing people’s decisions to shop for food online.$X_{i}^{^{\prime}}$ is a vector of control variables that are expected to affect dietary diversity.

In particular, $Z_{i}^{^{\prime}}$ and $X_{i}^{^{\prime}}$ are allowed to overlap, and $Z_{i}^{^{\prime}}$ usually contains all the variables included in $X_{i}^{^{\prime}}$ and at least one additional variable that serves as an instrumental variable (IV). In this study, a binary variable identifying whether a household’s relatives or friends use online shopping (1 = Yes and 0 = No) is employed as the IV. Peers have a tendency to emulate one another (Chen 2008). Furthermore, individuals faced with conflicting information tend to trust the recommendation and endorsements of people they know. Xu et al. (2017) showed that informational incentives between peers and the social influence they exert on one another affect individuals’ online shopping behaviors. Thus, it is reasonable to assume that households whose relatives or friends shop online would also be more likely to shop online. The falsification tests presented in Table A1 of Appendix confirm the validity and effectiveness of the employed IV (Pizer 2016; Chen et al. 2019; Li et al. 2020).

**Table 1:** Variable definitions and summary statistics

Variables	Definition	Mean (S.D.)
*Dependent variables*		
Dietary diversity	The number of food items consumed by a household in the last 72 h (2–12)	6.57 (2.16)
Online food shopping	1 if respondent purchased food items online, 0 otherwise	0.18 (0.38)
*Independent variables*		
Age	Age of respondent (years)	52.53 (12.74)
Gender	1 if respondent was male, 0 otherwise	0.40 (0.49)
Education	Education level of respondent (years)	7.78 (4.15)
Household size	Number of members residing in a household (persons)	4.14 (1.75)
Land size	Size of land cultivated by a household (mu) ^a^	4.67 (31.68)
Oven ownership	1 if household owned a microwave oven, 0 otherwise	0.30 (0.46)
Heath knowledge	1 if respondent knew “The Dietary Guidelines for Chinese Residents”, 0 otherwise	0.14 (0.34)
Motor ownership	1 if household owned a motorcycle, 0 otherwise	0.24 (0.42)
Distance to credit	Distance to the nearest informal credit sources (e.g., friends and relatives) or formal credit sources (e.g., banks) (km)	2.98 (6.26)
Distance to market	Distance to the nearest food market (km)	2.57 (2.90)
Shandong	1 if household was in Shandong province, 0 otherwise	0.25 (0.43)
Guangxi	1 if household was in Guangxi province, 0 otherwise	0.25 (0.43)
Henan	1 if household was in Henan province, 0 otherwise	0.24 (0.43)
Sichuan	1 if household was in Sichuan province, 0 otherwise	0.26 (0.44)
IV	1 if household’s relatives or friends used online shopping; 0 otherwise	0.59 (0.49)
Observations		947

^a^1 mu = 1/15 hectare. S.D. refers to the standard deviation

Because $Y_{1i}$ and $Y_{0i}$, the dietary diversity scores, are measured as count variables, the Poisson regression models should be used to estimate Eqs. (2a) and (2b). Under the normality assumption, the joint probability $f_{j} \left( {Y_{i} ,{ }T_{i} \left| {X_{i} } \right.,{ }Z_{i} } \right)$ for a Poisson distribution can be written as follows (Hasebe 2020)^2^:

3 $$f_{j} \left( {Y_{i} , T_{i} \left| {X_{i} } \right., Z_{i} } \right) = \mathop \smallint \limits_{ - \infty }^{\infty } f_{j} \left( {Y_{i} \left| {X_{i} } \right., \eta_{j} } \right){\Phi }\left\{ {\frac{{\left( {2T_{i} - 1} \right)\left( {Z_{i}^{^{\prime}} \alpha_{i} + \rho_{j} \sigma_{j}^{ - 1} \eta_{j} } \right)}}{{\sqrt {1 - \rho_{j}^{2} } }}} \right\}\phi \left( {\eta_{j} } \right){\text{d}}\eta_{j}$$

where ${\Phi }\left\{ \cdot \right\}$ and $\phi$ ($\cdot$) are the cumulative distribution function and probability density function of standard normal distribution, respectively. $\rho_{j}$ represents the correlation coefficient between error terms $\varepsilon_{i}$ and $\eta_{ji}$ for $j = 0,{ }1$. $\sigma_{j}$ is the standard deviation of $\eta_{ji}$. In particular, $f_{j} \left( {Y_{i} \left| {X_{i} } \right., \eta_{j} } \right)$ in Eq. (3) can be defined as follows:

4 $$f_{j} \left( {Y_{i} \left| {X_{i} } \right., \eta_{ji} } \right) = \frac{{{\text{exp}}\left( {X_{i}^{^{\prime}} \beta_{ji} + \eta_{ji} } \right)^{{Y_{i} }} {\text{exp}}\left\{ { - {\text{exp}}\left( {X_{i}^{^{\prime}} \beta_{ji} + \eta_{i} } \right)} \right\}}}{{Y_{i} !}}$$

The Stata command “*escount*” estimates the parameters $\vartheta = \left( {\alpha^{\prime},{ }\beta_{1}^{^{\prime}} ,{ }\beta_{0}^{^{\prime}} ,{ }\sigma_{1} ,{ }\sigma_{0} ,{ }\rho_{1} ,{ }\rho_{0} } \right)$. If $\rho_{j}$ ($j = 0,{ }1$) is statistically significant, this would suggest the presence of selection bias arising from unobserved factors. After estimating the parameter vectors $\alpha^{\prime},{ }\beta_{1}^{^{\prime}}$ and $\beta_{0}^{^{\prime}}$ for the selection equation and the two outcome equations, one can calculate the treatment effects of online food shopping. We were interested in estimating the average treatment effects on the treated (ATT) and average treatment effects on the untreated (ATU). These can be expressed as follows:

5a $$\mu_{{{\text{ATT}}}} = E\left[ {Y_{1} - Y_{0} \left| {X, T_{i} = 1} \right.} \right] = E\left[ {Y_{1} - Y_{0} \left| {X, \varepsilon_{i} > Z_{i}^{^{\prime}} \alpha_{i} } \right.} \right]$$

5b $$\mu_{{{\text{ATU}}}} = E\left[ {Y_{1} - Y_{0} \left| {X, T_{i} = 0} \right.} \right] = E\left[ {Y_{1} - Y_{0} \left| {X, \varepsilon_{i} \le Z_{i}^{^{\prime}} \alpha_{i} } \right.} \right]$$

The expected outcome variables (i.e., dietary diversity scores) for randomly chosen online food shoppers ($j = 1$) and non-shoppers ($j = 0$), conditional on the treatment status, were estimated using Eq. (6):

6 $${\text{E}}\left( {Y_{j} \left| {X, T} \right.} \right) = {\text{exp}}\left( {X_{i}^{^{\prime}} \beta_{j} + \sigma_{j}^{2} /2} \right)\frac{{{\Phi }\left\{ {\left( {2T - 1} \right)\left( {\rho_{j} \sigma_{j} + Z_{j}^{^{\prime}} \alpha_{i} } \right)} \right\}}}{{{\Phi }\left\{ {\left( {2T - 1} \right)\left( {Z_{j}^{^{\prime}} \alpha_{i} } \right)} \right\}}}$$

Finally, ATT and ATU were derived using the Stata command “*teescount*” (Hasebe 2020):

7a $$\hat{\mu }_{{{\text{ATT}}}} = \frac{{\mathop \sum \nolimits_{i}^{N} \left( {T_{i} } \right)\mu_{{{\text{ATT}}}} }}{{\mathop \sum \nolimits_{i}^{N} T_{i} }}$$

7b $$\hat{\mu }_{{{\text{ATU}}}} = \frac{{\mathop \sum \nolimits_{i}^{N} \left( {1 - T_{i} } \right)\mu_{{{\text{ATU}}}} }}{{\mathop \sum \nolimits_{i}^{N} (1 - T_{i} )}}$$

## Data, key variables, and descriptive statistics

### Data

The data for this study were drawn from a survey on rural households’ food expenditure and nutrition intake conducted by the Institute of Food and Nutrition Development, Ministry of Agriculture and Rural Affairs of the People’s Republic of China. The survey utilized a multistage random sampling strategy to select rural households from Shandong, Guangxi, Henan, and Sichuan provinces between June and November 2019. In the first stage, the four provinces were chosen based on their geographical locations and economic development levels. For example, Shandong and Henan provinces are in Eastern and Central China, respectively, while Sichuan and Guangxi are located in Western China; disposable incomes per capita of rural households in these four provinces were 18.8, 16,1, 15.9, and 14.8 thousand yuan, respectively, in 2020.^3^ In the second and third stages, five counties from each selected province and then four villages from each selected county were randomly chosen. The following counties were selected: Gaotang, Chengyang, Shouguang, Lanling, and Laiwu in Shandong; Haicheng, Yizhou, Qingxiu, Guanyang, and Teng in Guangxi; Ye, Huaibin, Lingbao, Huaiyang, and Anyang in Henan; and Chongzhou, Lushan, Xuyong, Zizhong, and Langzhong in Sichuan. In the final stage, between 12 and 14 rural farmers from each selected village were randomly chosen, resulting in a sample of 1,051 households. To clean the data, we dropped observations with missing information or outliers. For example, households with zero members or exceptionally high food intake were dropped. The final sample comprised 947 observations.

The selected rural households were interviewed face-to-face by pre-trained undergraduate and postgraduate students from local universities. We used a structured and pre-tested questionnaire to collect information on households’ dietary patterns by asking questions about the food consumed over the previous three days and the previous year. The respondents were asked to provide information on the names and quantities of the food items they consumed, whether they shopped for food online, and how knowledgeable they were about nutrition and food quality. The questionnaire also comprised questions on household members’ age, gender, education, weight, height, marital status, types of employment, and religion. Although household heads were interviewed and were the primary respondents, other members also helped complete the survey.

To ensure the accuracy of the information, the enumerators asked the household member in charge of preparing meals to answer the questions in the diet and food consumption module. Since it can be difficult for respondents to recall detailed food consumption for each meal over the previous three days (72-h recall), the dietary information was collected in two steps. First, the enumerators recorded the information related to household food consumption for breakfast, lunch, and dinner over the past 48 h (two days). Then, on the following day, the enumerators re-visited the same households to gather their food consumption information for the past 24 h. The two-step collection procedure results in a better recall, and thus, more accurate data. The proportion of consumed food to leftover food was also calculated to estimate the actual food intake rather than the food purchased.

### Key variables

The outcome variable in our study was dietary diversity, measured using the household dietary diversity scores. The dietary diversity score was defined as the number of food groups consumed by rural households in the last 72 h. Consistent with previous studies (Kennedy et al. 2011; Desta et al. 2019; Muthini et al. 2020; Argaw et al. 2021; Olabisi et al. 2021; Nikiema and Sakurai 2021), the 12 food groups included (1) cereals, (2) roots and tubers, (3) legumes, (4) vegetables, (5) edible fungi, (6) fruits, (7) nuts and seeds, (8) livestock meat, (9) poultry meat, (10) milk and milk products, (11) eggs, and (12) fish and seafood. The dietary diversity score ranged from 2 to 12 because each food group was counted once if it was consumed. The dietary diversity score is a simple but useful proxy for measuring households’ food security and access to various kinds of food. A higher dietary diversity score signifies a more varied diet.

The treatment variable was online food shopping. Online food shopping was defined as a dichotomous variable and assigned a value of one if rural households had purchased food via online shopping websites and apps (e.g., Tmall; Taobao; and JD) and zero otherwise. This definition is consistent with previous studies on online shopping behaviors (Hao et al. 2020; Zheng et al. 2020; Gao et al. 2020; Zheng and Ma 2021a). Following the literature on online food shopping (e.g., Heng et al. 2018; Wang and Somogyi 2018; Bryła 2018; Balachandran et al. 2020; Lee et al. 2020; Liu and Lin 2020; Alaimo et al. 2020; Chang and Meyerhoefer 2021; Ma et al. 2022) and dietary diversity (e.g., Koppmair et al. 2017; Miyamoto et al. 2019; Aweke et al. 2020; Olabisi et al. 2021), we selected control variables that captured individual, demographic, household, and regional characteristics. Specifically, the variables representing the age, gender, and education level of the respondents, household size, land size, oven ownership, health knowledge, motor ownership, distance to credit, distance to market, and regional dummies were used in the regression models.

### Descriptive statistics

Table 1 presents the summary statistics of the variables used in the analysis. The average dietary diversity score was 6.57 out of 12, suggesting that sampled households consumed, on average, seven types of food groups in the last 72 h. It shows that only 18% of households purchased food items online. The average age of the respondents was 53 years, and 40% of them were male. The average education of sampled respondents was 7.78 years. The average household size was four, while the average land size was 4.67 mu (1 mu = 1/15 hectare). On average, the distances to credit sources and the nearest food market were 2.98 and 2.57 km, respectively. Respondents from Shandong, Guangxi, Henan, and Sichuan provinces accounted for 25%, 25%, 24%, and 26%, respectively, suggesting that the observations were evenly distributed across the four provinces.

The food group categories to calculate dietary diversity are reported in Table 2. Sampled households purchased various fresh and processed food items online. It shows that all households consumed cereals, and for each household member, the average intake of cereals was 450.5 g per day. Similarly, almost all households (99.8%) consumed vegetables, and the average vegetable intake per capita was 365.5 g/day. Sampled households consumed more livestock meat than poultry meat. The proportions of households that consumed livestock and poultry meat were 71.2% and 32.2%, respectively. As shown in the third column in Table 2, the average per capita intake of livestock meat and poultry meat was 83.7 and 32 g/day, respectively. The least consumed food types were milk and milk products, nuts and seeds, and edible fungi—these were consumed by 30.5%, 21.5%, and 20.5% of the sampled households, respectively.

**Table 2:** Descriptive statistics of food groups that consumed by households

Groups	Food types	Food intake per capita (gram/day)	% of households
Cereals	Rice, wheat, maize, and other cereals (e.g., oats, barley)	450.5	100.0
Roots and tubers	Potatoes, red kumara, purple kumara, and other roots and tubers	54.0	57.3
Legumes	Soybeans, tofu, bean sprouts and other legumes	41.9	60.2
Vegetables	Cauliflower, leaf vegetable, fruit vegetable, and other vegetables	365.5	99.8
Edible fungi	Oyster mushrooms, pleurotus eryngii, flammulina velutipes, and other edible fungi	4.4	20.5
Fruits	Melons, oranges, and other fruits	142.6	56.2
Nuts and seeds	Peanuts, walnuts, pine nuts, and sunflower seeds, and other nuts	9.5	21.5
Livestock meat	Beef, lamb, and pork, and other livestock meat	83.7	71.2
Poultry meat	Chicken, duck, goose, and other poultry meat	32.0	32.2
Milk and milk products	Milk, milk powder, cheese, yogurt, and other milk products	8.5	30.5
Eggs	Eggs and other egg foods	44.8	73.8
Fish and seafood	Fish, shrimp, and other seafood	44.3	33.6

Table 3 shows the mean differences in the outcome variable and control variables for online food shoppers and non-shoppers. The average dietary diversity for online food shoppers and non-shoppers was 7.33 and 6.40, respectively. The mean difference (0.93) was statistically significant at the 1% level. This suggests that online food shopping was associated with greater dietary diversity. The significant mean differences in the control variables (e.g., age, gender, education level, and health knowledge of the respondents) indicate that online food shoppers and non-shoppers were systematically different. For example, compared with non-shoppers, online food shoppers were younger, better educated, more likely to own a micro-oven, and lived closer to credit sources and food markets. These systematic differences between online shoppers and non-shoppers reinforce our suspicion concerning self-selection bias.

**Table 3:** Mean differences of the variables between online food shoppers and non-shoppers

Variables	Online food shoppers	Non-shoppers	Mean differences
Dietary diversity	7.33 (1.89)	6.40 (2.18)	0.93***
Age	41.16 (11.16)	55.04 (11.66)	− 13.88***
Gender	0.33 (0.47)	0.42 (0.49)	− 0.09**
Education	11.32 (4.18)	7.01 (3.71)	4.32***
Household size	4.29 (1.40)	4.11 (1.82)	0.18
Land size	7.42 (68.76)	4.06 (13.64)	3.36
Oven ownership	0.54 (0.50)	0.25 (0.43)	0.29***
Heath knowledge	0.30 (0.46)	0.10 (0.30)	0.20***
Motor ownership	0.27 (0.44)	0.23 (0.42)	0.04
Distance to credit	2.16 (2.28)	3.16 (6.82)	− 1.01*
Distance to market	2.10 (2.32)	2.67 (3.00)	− 0.57**
Shandong	0.21 (0.41)	0.26 (0.44)	− 0.05
Guangxi	0.31 (0.46)	0.23 (0.42)	0.08**
Henan	0.31 (0.46)	0.23 (0.42)	0.08**
Sichuan	0.17 (0.38)	0.28 (0.45)	− 0.11***
IV	0.98 (0.13)	0.51 (0.50)	0.48***
Observations	171	776	

*p < 0.10, **p < 0.05, and ***p < 0.01. Standard deviation is presented in parentheses

**Fig. 1:**
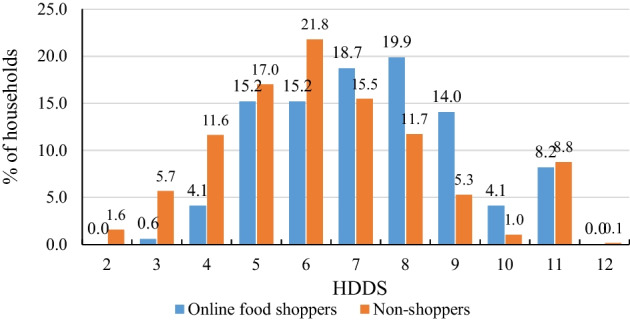
Distributions of dietary diversity scores by online food shopping status

Figure 1 illustrates the distribution of dietary diversity scores for online food shoppers and non-shoppers, providing further insights into the systematic differences between the two groups of shoppers. Households with dietary diversity scores of 8 and 7 accounted for the largest proportion of online food shoppers, representing 19.9% and 18.7%, respectively. In comparison, those with dietary diversity scores of 6 and 5 were the most prevalent among non-shoppers, accounting for 21.8% and 17.0%, respectively. We also found notable differences among households with low dietary diversity (i.e., those with dietary diversity scores < 5). In each case, the proportion of online food shoppers was lower than that of non-shoppers, suggesting once again that online food shopping was associated with greater dietary diversity.

## Empirical results

### Determinants of online food shopping

Using the MLE, we jointly estimated the selection Eq. (1) and the outcome Eqs. (2a) and (2b). Table 4 presents the empirical results. The correlation coefficients $\rho_{0}$ and $\rho_{1}$ (lower part of Table 4) were statistically significant, indicating the presence of selection bias due to unobserved factors and justifying the appropriateness of using the ESC model (Hasebe 2020). Notwithstanding the joint estimation, it is instructive to discuss the determinants of online shopping first. The coefficients presented in column 2 of Table 4 correspond to the selection equation. These were obtained from a standard probit model and thus illuminated the predicted probabilities of rural households to shop for food online.

**Table 4:** Impact of online food shopping on dietary diversity: ESC model estimations

Variables	Selection (coefficients)	Dietary diversity	
		Online food shoppers (coefficients)	Non-shoppers (coefficients)
Age	− 0.039 (0.007)***	− 0.003 (0.002)*	− 0.004 (0.001)***
Gender	− 0.181 (0.132)	− 0.006 (0.033)	− 0.010 (0.024)
Education	0.057 (0.020)***	0.011 (0.004)***	0.007 (0.003)**
Household size	− 0.028 (0.043)	− 0.026 (0.011)**	0.006 (0.007)
Land size	− 0.000 (0.001)	0.000 (0.000)*	− 0.001 (0.001)
Oven ownership	0.477 (0.129)***	0.094 (0.036)***	0.040 (0.025)
Heath knowledge	0.569 (0.158)***	0.071 (0.033)**	0.046 (0.030)
Motor ownership	− 0.046 (0.148)	− 0.013 (0.039)	− 0.002 (0.026)
Distance to credit	− 0.021 (0.013)	− 0.023 (0.008)***	− 0.002 (0.002)
Distance to market	− 0.009 (0.026)	0.037 (0.008)***	− 0.002 (0.004)
Shandong	− 0.068 (0.188)	− 0.075 (0.056)	− 0.047 (0.030)
Guangxi	0.005 (0.188)	− 0.236 (0.054)***	− 0.256 (0.034)***
Henan	0.180 (0.179)	− 0.329 (0.055)***	− 0.402 (0.037)***
IV	1.328 (0.258)***		
Constant	− 0.644 (0.526)	2.178 (0.131)***	2.163 (0.093)***
${\text{Ln}}\sigma _{1}$		− 5.806 (1.942)***	
$\rho_{1}$		− 0.898 (0.064)***	
${\text{Ln}}\sigma_{0}$			− 6.559 (3.084)**
$\rho_{0}$			− 0.907 (0.104)**
Observations	947	171	776

*p < 0.10, **p < 0.05, and ***p < 0.01. The reference province is Sichuan. Robust standard errors are presented in parentheses

The coefficient of age was negative and significant, indicating that older individuals were less likely to shop for food online. This is to be expected, as older individuals, relative to younger ones, tend to be less competent users of ICTs such as computers, smartphones or tablets (Penard et al. 2015; Ma and Wang 2020). Thus, older individuals are less trustful of ICTs and habituated to traditional food shopping. In contrast, the coefficient of education was positive and significant, suggesting that the higher the education level, the greater the predicted probability of shopping for food online. This finding is consistent with Zheng and Ma (2021b). They noted that better-educated individuals are more likely to have the requisite skills to navigate E-commerce platforms and make purchases. Owning a microwave oven also increased the predicted probability that a household shopping for food online. This reflects the synergistic effects of online food shopping and using microwave ovens for cooking meals. For example, purchasing pre-packaged frozen meals online and cooking them in the oven saves time on two accounts: one need not travel to purchase the ingredients or spend time preparing the meals at home. Our results coincide with Asfaw (2011) and Rupa et al. (2019), who showed that microwaves allowed people to consume a more comprehensive array of food products. The positive and significant coefficient of health knowledge signifies that individuals who knew the dietary guidelines for Chinese residents were more likely to shop for food online than those who did not know the guidelines. This is reassuring, considering that access to supermarkets via online shopping may lead to poor dietary choices (Asfaw 2008). Lastly, the coefficient of the IV was also positive and significant—having friends or relatives who bought food online increased the predicted probability of individuals doing the same. This points to the role of word-of-mouth in promoting the uptake of online shopping. Friends and relatives may inform and educate people on the potential benefits of online shopping, instilling confidence and trust in the safety and convenience of this mode of shopping. It bears emphasizing that the IV was only used in the first-stage selection equation to identify the model correctly.

### Determinants of dietary diversity

In this section, we turn our attention to the second-stage results obtained using Poisson regression models. Two outcome equations were estimated, one for those who used online food shopping and the other for those who did not. The results are presented in the last two columns of Table 4, revealing that more factors affected the dietary diversity of online food shoppers than of those who did not shop for food online. Furthermore, the significant coefficients for the two groups had similar magnitudes.

The coefficients of age were statistically significant, indicating that older people had a lower dietary diversity. Education significantly increased dietary diversity. However, the effect of education on dietary diversity was negligible. Online food shoppers living in larger households tended to have lower dietary diversity than those living in smaller ones. This contradicts previous studies and may seem counterintuitive (Rupa et al. 2019; Chegere and Stage 2020). After all, it is reasonable to assume that having more members in a household would result in the demand for a greater variety of food products: food is purchased with due consideration to each household member’s tastes and preferences, and variation in individuals’ taste and preference is to be expected. However, larger households may purchase food items that are palatable to all household members, leaving few options that lie at the intersection of largely different preferences, thereby reducing dietary diversity across the households. It may also be the case that larger households having lower per capita resources at their disposal economize by purchasing a few food items in bulk. Our results are more in line with this reasoning. In fact, even at a constant per capita total expenditure, per capita demand for food may decrease (Deaton and Paxson 1998). This, too, may lead to lower dietary diversity. Of course, each household member may prefer specific food items regardless of the available variety—having more members in the household may not alter anyone’s dietary choices.

The dietary diversity score for online shoppers owning microwave ovens was significantly higher than those who did not own them. As noted in Sect. 4.1, microwaves allow households to consume a greater variety of food products, including frozen meals, baked items, and ready-to-eat meals that require only heating or minimal preparation. Being more knowledgeable about dietary guidelines increased dietary diversity. Individuals who knew the dietary guidelines for Chinese residents had a higher dietary diversity score than those who did not. Prepared by the Chinese Nutrition Society, the guidelines are presented in a ‘Food Guide Pagoda’ that comprises five levels representing different food groups. Two additional visualizations have been developed to promote the consumption of balanced diets (FAO 2021). Specifically, people are encouraged to consume various foods such as vegetables, milk, soybean, fish, poultry, eggs, and lean meat; cereals are the recommended staple. Our results affirm the benefits of developing and disseminating dietary guidelines to the Chinese people. These results are consistent with other studies showing the benefits of nutrition education and interventions on biomarkers and diets (Hamidianshirazi et al. 2022).

Proximity to credit sources increased the dietary diversity of online food shoppers. To be clear, we considered formal and informal credit sources. The latter included friends and family. Living near them may promote social eating and, in turn, increase dietary diversity. Moreover, accessibility to credit may instill financial security, causing people to spend relatively freely on food, thereby increasing their dietary diversity. On the other hand, an increase in the distance to the nearest market increased the dietary diversity score for online food shoppers.

Geographical factors were significant for both groups of shoppers. Residents of Sichuan province had higher dietary diversity than those of Guangxi and Henan provinces. Nevertheless, there was no difference in the dietary diversity of those living in the Shandong and Sichuan provinces. Relative to the dietary diversity score of online food shoppers living in the Sichuan province, the dietary diversity scores of those living in the Guangxi and Henan provinces were lower. These differences were larger among individuals who did not buy food online. This result is suggestive. Online food shopping has a role in bridging the dietary diversity gaps across provinces.

### Incidence rate ratios (IRRs) of Poisson regression models

Because the interpretation of the coefficients estimated from Poisson regression models is not straightforward, we also calculate IRRs and present them in Table 5. These are exponential transformations of the Poisson regression coefficients and render direct and meaningful interpretations of the link between dietary diversity scores and various factors of interest, that is, ${\text{IRR}} = \exp \left( {{\text{coefficient}}} \right)$ (Erdogdu 2013; Zhang et al. 2020; Ma and Wang 2020). More specifically, should an independent variable increase by one unit, the dependent variable (i.e., dietary diversity score) would decrease by $\left[ {\left( {1 - {\text{IRR}}} \right)*100\% } \right]$ when the IRR is smaller than one and increase by $\left[ {\left( {{\text{IRR}} - 1} \right)*100\% } \right]$ when the IRR is greater than one.

**Table 5:** Determinants of dietary diversity for online food shoppers and non-shoppers: IRR estimates

Variables	Dietary diversity	
	Online food shoppers (IRRs)	Non-shoppers (IRRs)
Age	0.997*	0.996***
Gender	0.994	0.990
Education	1.011***	1.007**
Household size	0.974**	1.006
Land size	1.000*	0.999
Oven ownership	1.099***	1.041
Heath knowledge	1.074**	1.047
Motor ownership	0.987	0.998
Distance to credit	0.977***	0.998
Distance to market	1.038***	0.998
Shandong	0.928	0.954
Guangxi	0.790***	0.774***
Henan	0.720***	0.669***
Constant	8.829***	8.697***
Observations	171	776

*p < 0.10, **p < 0.05, and ***p < 0.01. The reference province is Sichuan. Robust standard errors are presented in parentheses

The IRRs of age for online shopping users and non-users were 0.997 and 0.996 (both smaller than 1), respectively. The findings suggest that a one-year increase in age decreased the dietary diversity score by 0.3% for online food shoppers and 0.4% for non-shoppers. The IRRs for education for online shoppers and non-shoppers were 1.011 and 1.007 (both bigger than 1), respectively. The findings suggest that a one-year increase in education increases dietary diversity score by 1.1% for online food shopping users and 0.7% for non-users. Furthermore, their dietary diversity scores declined by 2.6% for every additional household member. The dietary diversity scores for online shoppers owning microwave ovens were 9.9% greater than those who did not own them. As the distance to credit increased by one kilometer, the dietary diversity score decreased by 2.3% and compared with online food shoppers in Sichuan, those in Guangxi and Henan had 21% and 28% lower dietary diversity scores, respectively.

### Treatment effects of online food shopping

We calculate the ATT and ATU using Eqs. (7a) and (7b), respectively, to provide further insights into how online food shopping affects dietary diversity. The results are presented in Table 6. The estimated ATT was 0.501, suggesting that online food shopping increased the dietary diversity score by 7.34%. The estimated ATU, 0.378, was also statistically significant, indicating that those who did not shop for food online would have increased their dietary diversity score by 5.91% had they shopped for food online. As a robustness check, we also estimated the impact of online food shopping on dietary diversity using the PRETE model and IPWRA estimator. The results (Table A2 in Appendix) show that online food shopping increased dietary diversity by around 7–9%, very close to the percentage change (7.34%) estimated by the ESC model. The findings confirmed the robustness of the estimated effects. Overall, our estimates confirmed that online food shopping boosted dietary diversity among Chinese rural households. Higher dietary diversity may contribute positively and meaningfully to the health outcomes of rural Chinese.

**Table 6:** Average treatment effects of online food shopping on dietary diversity: ESC model estimates

Outcome	Mean outcomes		ATT	Change
	Online food shoppers (Actual)	Non-shoppers (Counterfactual)		
Dietary diversity	7.327 (1.232)	6.826 (1.241)	0.501 (0.190)***	7.34%

	Mean outcomes			
	Online food shoppers (Counterfactual)	Non-shoppers (Actual)	ATU	Change
Dietary diversity	6.778 (1.199)	6.401 (1.007)	0.378 (0.242)***	5.91%

***p < 0.01. Robust standard errors are presented in parentheses

ATT: average treatment effects on the treated, ATU: average treatment effects on the untreated

### Impacts of online food shopping on food consumption by types

In the analysis discussed above, we measured dietary diversity as a count variable comprising 12 food groups. Here, we investigate how online food shopping affected rural households’ consumption of different food types using the endogenous switching regression (ESR) model.^4^ The results (Table A3 in Appendix) show that online food shoppers are less likely to consume roots and tubers and vegetables compared with non-shoppers. However, relative to non-shoppers, online food shoppers consume more edible fungi, fruits, nuts and seeds, livestock meat, poultry meat, milk and milk products, eggs, and fish and seafood. Online food shopping did not significantly impact rural households’ consumption of cereals and legumes.

## Conclusions and policy implications

This study investigated the factors influencing rural households’ decisions to shop for food online and analyzed the impact of online food shopping on dietary diversity. Noting that people choose to shop online, we formally tested for and confirmed selection bias. We utilized a newly developed endogenous switching regression designed to model variations in count-dependent variables while accounting for selection bias.

The ATT showed that online food shoppers experienced a 7.34% increase in dietary diversity due to online food shopping. Furthermore, the ATU suggested that the dietary diversity of those who did not shop for food online would have increased by 5.91% had they shopped for food online. The results derived from the PRETE and IPWRA models confirmed the robustness of these findings. We found that education, asset ownership, and knowledge of the government’s dietary guidelines were positively associated with rural households’ adoption of online food shopping, while age was negatively associated with the same. Regarding the factors influencing the dietary diversity of online food shoppers, we found that it was positively associated with education, the size of one’s farm, asset ownership, knowledge of the government’s dietary guidelines, and distance to the nearest food market. In contrast, it was negatively associated with age, household size, and distance to credit sources. The dietary diversity of those who did not shop for food online was negatively affected by age but positively affected by education.

Our findings point to the benefits of harnessing the ubiquity and growth of E-commerce to improve the uptake of online food shopping, which may translate into increased dietary diversity in rural China. Two impediments to achieving this ought to be addressed. The first has to do with access to and familiarity with technology. Because not all rural residents can access the requisite technologies (e.g., the Internet, digital shopping carts, and online payment platforms) and have the know-how to shop online, the government should work with Internet service providers to ensure access to high-quality, affordable Internet in rural areas; it should also invest in training initiatives designed to help rural Chinese learn digital technologies. The second concerns the awareness of what constitutes a healthy diet and the consequences of eating different kinds of food. Our results showed that knowing the government’s dietary guidelines was associated with greater dietary diversity, pointing to the importance of disseminating dietary information among rural residents. Educational programs and informational seminars may be organized to equip rural households with the skills and knowledge to adopt healthy eating habits. Nutritional education has proven successful in changing diets and managing health conditions.

Collecting timely and accurate information is critical to designing sound policies and initiatives to positively influence people’s dietary habits; it is also challenging. Online food shopping can help overcome this challenge by providing accurate real-time data on people’s buying behaviors. These data can be analyzed using big data techniques, and actionable insights can be gathered to design policies to affect dietary changes. Online food shopping may lend itself well to gamification, which, in turn, may promote healthy eating habits. For example, grocers, employers, and governments may award points to shoppers for purchasing healthy food items and dock points for purchasing unhealthy ones—the shoppers may redeem these points for cash or apply them towards food discounts and lowering insurance premiums. The technology company IBM has used similar strategies to influence dietary patterns in the workplace. Online food shopping could play a foundational role in enabling such incentive-based initiatives—helping rural Chinese migrate to online food shopping may help the government achieve long-term public health goals by harnessing the power of data.

This study measured online food shopping as a dichotomous variable and thus presented a partial view of the associations between online food shopping and dietary divert. Thus, future studies can build upon our findings by studying the associations between online food shopping and consumption frequency and expenditure.

## Data Availability

The data that support the findings of this study are available from Hongyun Zheng upon request.

## Appendix

Tables 7, 8, 9.

**Table 7:** Falsification test of instrument variable

Outcome variables	Statistics
Dietary diversity	*χ*^*2*^ = 1.54; *p*-value = 0.214
Online food shopping	*χ*^*2*^ = 30.86***; *p*-value = 0.0001

****p* < 0.01

**Table 8:** Average treatment effects of online food shopping on dietary diversity: Robustness check

Methods	Mean outcomes		ATT	Change
	Actual	Counterfactual		
PRETE model	7.327 (1.260)	6.849 (1.178)	0.478 (0.176)***	6.98%
IPWRA estimator	7.327 (1.228)	6.727 (1.116)	0.601 (0.001)***	8.93%

****p* < 0.01. Robust standard errors are presented in parentheses

ATT: Aaverage treatment effects on the treated, ETPR: Poisson regression with endogenous treatment effects, IPWRA: Inverse-probability-weighted regression adjustment

**Table 9:** Average treatment effects of online food shopping on food intake per capita: ESR model estimates

Groups	ATT	*t*-value
Cereals	10.153 (267.852)	0.496
Roots and tubers	− 17.093 (31.312)***	− 7.139
Legumes	− 0.008 (34.245)	− 0.003
Vegetables	− 36.399 (146.587)***	− 3.247
Edible fungi	32.840 (7.430)***	57.799
Fruits	554.097 (181.041)***	40.023
Nuts and seeds	52.165 (14.066)***	48.496
Livestock meat	421.279 (246.909)***	22.312
Poultry meat	7.021 (22.346)***	4.109
Milk and milk products	61.143 (18.436)***	43.368
Eggs	115.311 (24.744)***	60.940
Fish and seafood	37.448 (79.541)***	6.157

*** < 0.01. Robust standard errors are presented in parentheses

ATT: average treatment effects on the treated, ESR: endogenous switching regression model

## Footnotes

1 Dietary diversity is defined as the number of different foods or food groups consumed over a given reference period (Ruel 2003). In this study, dietary diversity refers to the number of food items a rural household consumes during 72 h preceding the survey.

2 The joint probability can be assumed to have either a Poisson distribution or a negative binomial (NB) distribution in the ESC model (Hasebe 2020). Because our dependent variables range between 2 and 12, the NB distribution is not appropriate.

3 In June 2020, 1 Chinese yuan was equivalent to 0.14 US dollars.

4 The ESR model estimates the effect of a binary treatment variable on a continuous outcome variable, such as the intake of different types of food (see, for example, Leng et al. 2020; Ma et al. 2020; Dsouza 2021).
